# The hypoxia inducible factor/erythropoietin (EPO)/EPO receptor pathway is disturbed in a rat model of chronic kidney disease related anemia

**DOI:** 10.1371/journal.pone.0196684

**Published:** 2018-05-08

**Authors:** Daniel Landau, Lital London, Inbar Bandach, Yael Segev

**Affiliations:** 1 Department of Pediatrics B, Schneider Children’s Medical Center of Israel, Petach Tikva, Israel; 2 Sackler School of Medicine, Tel Aviv University, Tel Aviv, Israel; 3 Shraga Segal Department of Microbiology and Immunology, Faculty of Health Sciences, Ben Gurion University of the Negev, Beer Sheva, Israel; University Medical Center Utrecht, NETHERLANDS

## Abstract

**Objectives:**

Anemia is a known driver for hypoxia inducible factor (HIF) which leads to increased renal erythropoietin (EPO) synthesis. Bone marrow (BM) EPO receptor (EPOR) signals are transduced through a JAK2-STAT5 pathway. The origins of anemia of chronic kidney disease (CKD) are multifactorial, including impairment of both renal EPO synthesis as well as intestinal iron absorption. We investigated the HIF- EPO- EPOR axis in kidney, BM and proximal tibia in anemic juvenile CKD rats.

**Methods:**

CKD was induced by 5/6 nephrectomy in young (20 days old) male Sprague-Dawley rats while C group was sham operated. Rats were sacrificed 4 weeks after CKD induction and 5 minutes after a single bolus of IV recombinant human EPO. An additional control anemic (C-A) group was daily bled for 7 days.

**Results:**

Hemoglobin levels were similarly reduced in CKD and C-A (11.4 ± 0.3 and 10.8±0.2 Vs 13.5±0.3 g/dL in C, p<0.0001). Liver hepcidin mRNA was decreased in CA but increased in CKD. Serum iron was unchanged while transferrin levels were mildly decreased in CKD. Kidney HIF2α protein was elevated in C-A but unchanged in CKD. Kidney EPO protein and mRNA levels were unchanged between groups. However, BM EPO protein (which reflects circulating EPO) was increased in C-A but remained unchanged in CKD. BM and proximal tibia EPOR were unchanged in C-A but decreased in CKD. Proximal tibial phospho-STAT5 increased after the EPO bolus in C but not in CKD.

**Conclusions:**

Compared to blood loss, anemia in young CKD rats is associated with inappropriate responses in the HIF-EPO-EPO-R axis: kidney HIF2α and renal EPO are not increased, BM and bone EPOR levels, as well as bone pSTAT5 response to EPO are reduced. Thus, anemia of CKD may be treated with additional therapeutic avenues beyond iron and EPO supplementation.

## Introduction

Anemia prevalence in chronic kidney disease (CKD) increases as disease worsens, reaching 73% in children with stage 4 disease [1]. The appearance of this complication is associated with increased morbidity and mortality, as well as quality of life impairment [2]. Anemia of CKD is thought to be due to impaired renal erythropoietin (EPO) synthesis, since the kidney’s tubulointerstitial cells are the main synthesis site for this hormone after birth [3]. Other factors that have been found to be impaired in CKD are iron homeostasis as well as deficiency of folic acid and vitamin B12. In addition, RBC life span is decreased in uremia [4].

EPO is a 30 kDa glycoprotein, which facilitates normal erythropoiesis by enhancing the proliferation and differentiation of bone marrow erythroblasts, as well as inhibiting apoptosis [5]. The expression of EPO is significantly upregulated by hypoxia inducible factor (HIF) under hypoxic conditions [6]. HIF-2α is a major regulator of EPO, while HIF-1α is not [7]. However, in CKD there is no clear inverse correlation between anemia degree and EPO serum levels [8], suggesting that other mechanisms are of importance beyond EPO deficiency.

The EPO receptor (EPOR) is a 65 kDa glycoprotein, which belongs to the class 1 cytokine receptor family. It is expressed on red blood progenitor cells, and plays (through EPO stimulation) a central role in the rapid expansion of erythroid progenitors in response to anemia and hypoxic stimuli in vivo [9]. EPOR activation by its ligand creates homodimers and starts a chain of signal transduction that involves JAK2 and STAT5 phosphorylation. The latter enters the nucleus and activates EPO responsive genes [10].

The use of synthetic EPO-like molecules (erythropoiesis stimulating agents—ESA) has brought a major change in the management of CKD anemia [11]. However, many patients remain unresponsive to ESA, even after the correction of iron deficiency. Increasing ESA doses in these cases may be associated with side effects [12]. In adults, administration of high doses of ESA have been associated with death [13]. The main clinical factors associated with ESA resistance include inflammation, iron deficiency and hyperparathyroidism [14].

We have previously shown a decrease in epiphyseal growth plate (EGP) GH receptor (GHR) signaling and vascularization in growth impaired CKD rats [15]. In addition, we have also shown an increase in renal inflammation (IL6, p-STAT3) in a 5/6 nephrectomy model of CKD [16]. Since both GHR and EPO-R signals are transduced through the JAK2-STAT5 pathway, the purpose of our study was to characterize the (renal) HIF- (renal) EPO- (bone marrow)- EPOR axis in anemic juvenile CKD rats.

## Materials and methods

### Animal experimentation

The experimental protocol was approved by the Ben Gurion University committee for animal experimentation, in adherence to the NIH Guidelines. Animals were housed in standard laboratory cages provided with normal rat chow (containing 200 mg/g iron and 0.7% phosphate) (Harlan Laboratories, Jerusalem, Israel) with free access to unlimited amount of tap water. No attempts at pair feeding were tried. In a first set of experiments, male Sprague-Dawley rats were divided into 2 groups: C vs. daily tail bleeding of 150 uL of blood per day for 7 days. Rats were then sacrificed after anesthesia with ketamine and xylazine, collecting: blood, kidney, proximal tibia (PT) (which includes the epiphyseal growth plate and the primary ossification center) and bone marrow aspirate.

In a second series of experiments Young (20 d) Sprague-Dawley rats were divided into 2 groups: C vs. CKD (by standard 5/6 nephrectomy). A single bolus of IV rhEPO (25 U/kg) was administered 5 minutes prior to sacrifice. This dose and injection regimen was chosen after a series of preliminary experiments conducted in control rats, entailing different doses and time of injection of rhEPO prior to sacrifice, to find the dosage and time interval that induce a submaximal increase in bone p-STAT5. Rats were sacrificed after 4 weeks from second surgery after anesthesia with ketamine and xylazine. Blood was drawn from the vena cava at sacrifice. Serum was separated and frozen at -80°C. The right PT was removed and frozen in -80°C for western blot analysis or real time PCR, while the left PT was fixed in 4% formalin for histological analysis. Kidney and a bone marrow aspirate were isolated for protein and mRNA analysis. Bone marrow cells were released by flushing of rat femur and tibia with cold sterile phosphate buffered saline (PBS). Cells were then pelleted and washed.

### Immunoassays

Blood and urine samples were collected and frozen at -80°C until analysis. Serum and urine biochemistries were analyzed by the Biochemistry laboratory of Soroka Medical Center (Beer-Sheva, Israel). Serum and urine creatinine concentrations were assessed using the standard Jaffe reaction. Urine albumin concentrations were measured using the "Microalbumin" method (Beckman-Coulter, CA, USA). Serum rat EPO (n = 7 in each group) was determined by specific enzyme-linked immunosorbent assay kit (Sigma-Aldrich, Saint louis, MO, USA) according to the manufacturer’s instructions. The low detection limit was 0.07 ng/mL and the intra- and inter-assay coefficients of variation were <10 and 12%, respectively.

### RNA extraction and real time-PCR

Total RNA was extracted from liver, kidney and bone marrow using the PerfectPure RNA Tissue kit (Gentra Systems, Minneapolis, MN, USA) and cDNAs were synthesized using high-capacity cDNA reverse transcription kit (Applied Biosystems, Foster City, CA, USA). Quantitative real time PCR (qPCR) assays were performed with power SYBR green PCR master mix (Applied Biosystems, Foster City, CA, USA) as previously described [15,17], using the ABI Prism 7300 Sequence detection System (Applied Biosystems, Foster City, CA). Primers for quantification of Hepcidin, HIF-2 α, EPO, EPOR and β-actin (Sigma-Aldrich, Rechovot, Israel) are summarized in (Table 1). Each sample was analyzed in triplicate in individual assays. The specificity of the reaction is derived by the detection of the melting temperatures (Tms) of the amplification products immediately after the last reaction cycle. The target genes expression value was calculated by the ΔΔct method after normalization with a housekeeping gene (β-actin). Bone marrow aspirates cells were first suspended in RNA Save solution (Biological Industries, Beit Haemek, Israel). The remaining analysis was similar to the above mentioned methods.

**Table 1:** PCR primers list.

Gene	Forward	Reverse
Hif-2α	TTGCGGGGGTTGTAGATG	ACTTGGACGCTCTGCCTATG
EPO	GGGGGTGCCCGAACG	GGCCCCCAGAATATCACTGC
EPOR	CTCATCTCACTGTTGCTGACTGTGC	GTGGGTGGTGAAGAGACCCTCAA
Hepcidin	CACGAGGGCAGGACAGAAGGCAAG	CAAGGTCATTGCTGGGGTAGGACAG
β-Actin	GGTCTCAAACATGATCTGGG	GGGTCAGAAGAATTCCTATG

### Western immunoblot analysis

Kidney, PT and bone marrow tissues were homogenized on ice with a polytron (Kinetica, Littau, Switzerland) in lysis buffer (50 mM Tris, pH 7.4, 0.2% Triton X-100) containing 20 mM sodium pyrophosphate, 100 mM NaF, 4 mM EGTA, 4 mM Na3VO4, 2 mM PMSF, 0.25% aprotinin and 0.02 mg/ml leupeptine. Extracts were centrifuged for 20 minutes at 17,000g at 4oC and the supernatants collected and frozen. The following antibodies were used for evaluation of the extracts: HIF-2 α (Novus Biologicals, Littleton, Colorado, USA). STAT5, EPO and EPOR (Santa Cruz Biotechnology, CA, USA), p-STAT5 (Tyr 694) (Cell signaling Technology Inc. Denvers, MA) and β-actin (MP Biomedical Solon, OH, USA). Homogenates were mixed with 5X sample buffer, boiled for 5 minutes, loaded in each gel lane and subjected to 7.5–10% SDS polyacrylamide gel, and electroblotted into nitrocellulose membranes. Blots were blocked for 1 hour in Tris buffered saline—Tween 20 (TBST) (0.05% Twin-20) buffer (10 mM tris, pH 7.4, 138 mM NaCl) containing 5% non-fat dehydrated milk, followed by overnight incubation with the antibodies diluted in TBST (0.05% Twin-20) containing 5% dry milk. The phosphorylated antibody was diluted in TBST (0.05% Twin-20) containing 5% BSA (MP Biomedical, Solon, OH, USA). After washing 3 times for 15 minutes in TBST (0.05% Twin-20), the blots were incubated with secondary antibodies conjugated with horseradish peroxidase for 1 hour at room temperature and then washed again 3 times. The band antibody was visualized by enhanced chemiluminescense (ECL; Amersham, Life Sciences Inc.) and exposed to Kodak-BioMax film (Eastman Kodak, Rochester NY, USA). Protein expression was quantitated densitometrically using Fluorchem software (Alpha-Innotech, California, USA). For bone marrow protein analysis, we used a RIPA buffer (Millipore, Billerica, USA) with protease inhibitor (Roche, Indianapolis, USA). The other steps of protein analysis were similar to the above mentioned methods.

### Immunohistochemistry

The left tibia was fixed in 4% formalin and 5-μm-thick longitudinal sections were cut. For EPOR immunohistochemistry, deparaffinized, rehydrated sections were treated with 3% H2O2 for 15 min at room temperature to block endogenous peroxidase activity. The sections were washed with PBS before blocking with 2.5% normal horse serum (Vector laboratories Inc., Burlingame, CA, USA) for 1 h followed by overnight incubation in primary mouse anti-rabbit EPOR antibody (Santa Cruz Biotechnology California, USA) diluted 1:100 in the blocking serum. After incubation in primary antibody, sections were washed in PBS. Then they were incubated in appropriately diluted biotinylated secondary antibody for 10 min, washed with PBS, followed by incubation in streptavidin-peroxidase for 10 min and washed in PBS. The sections were subsequently incubated with buffered substrate solution (pH 7.5) containing hydrogen peroxide and 3,3-diaminobenzidine chromogen solution (Vector laboratories Inc., Burlingame, CA, USA). Sections were then dehydrated and mounted with permount and examined by light microscope. For the image processing Cellsense Entry software (MATIMOP, Tel Aviv, Israel) was used.

### Statistical analysis

Values along the manuscript are presented as means +/- standard errors. Two-tailed unpaired Student’s t-tests were applied for comparison of two normally distributed groups. Comparisons between more than 2 normally distributed groups were made by one-way ANOVA. A p value < 0.05 was considered significant.

## Results

**Fig 1:**
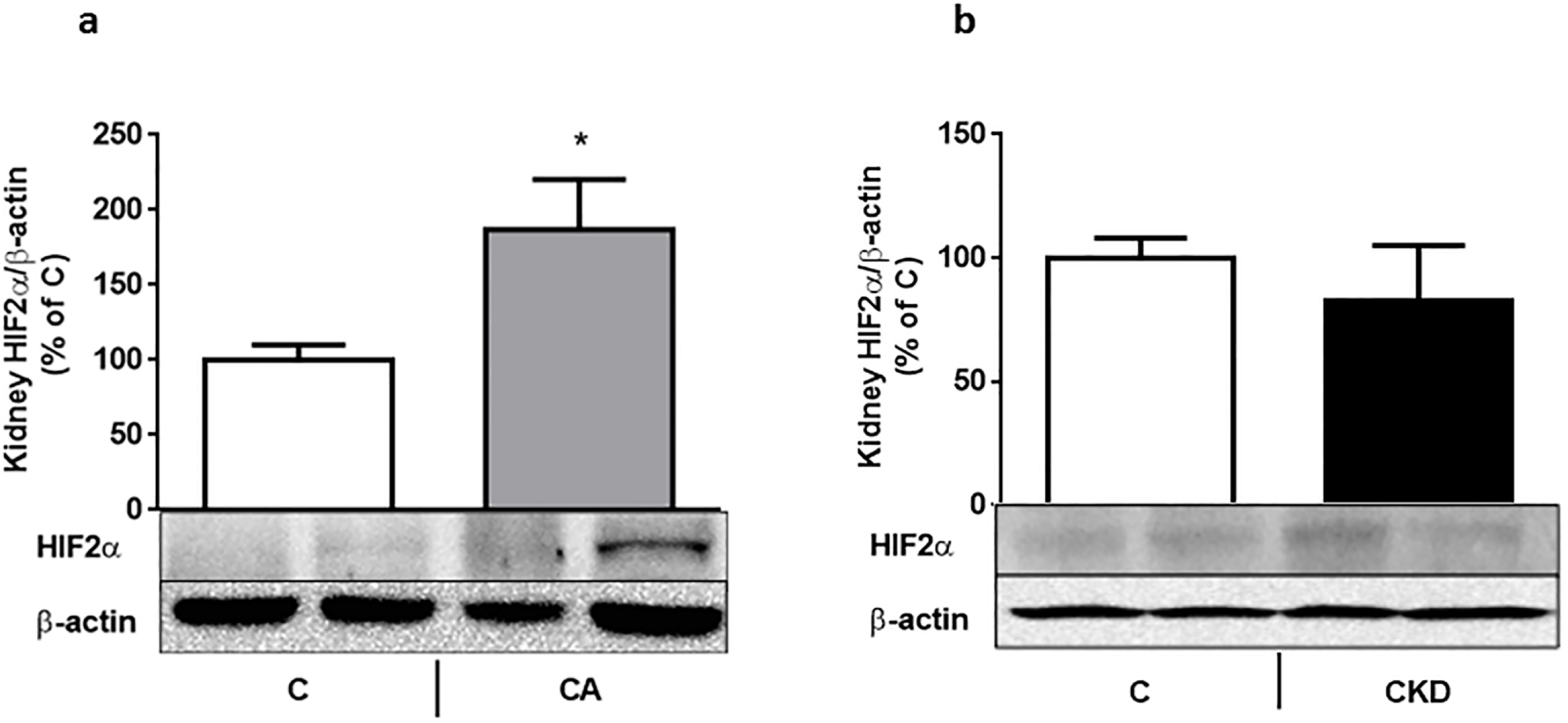
a-b: Kidney HIF2 α does not increase in uremic anemia. Kidney HIF2 α protein (as percentage of actin-corrected concentration) in control and 7 day-bled rats (Fig 1a) and in control and uremic (4 weeks after 5/6 nephrectomy) rats. * p<0.01). The lower panel in each figure shows a representative gel. 6–8 rats were used in each group.

**Fig 2:**
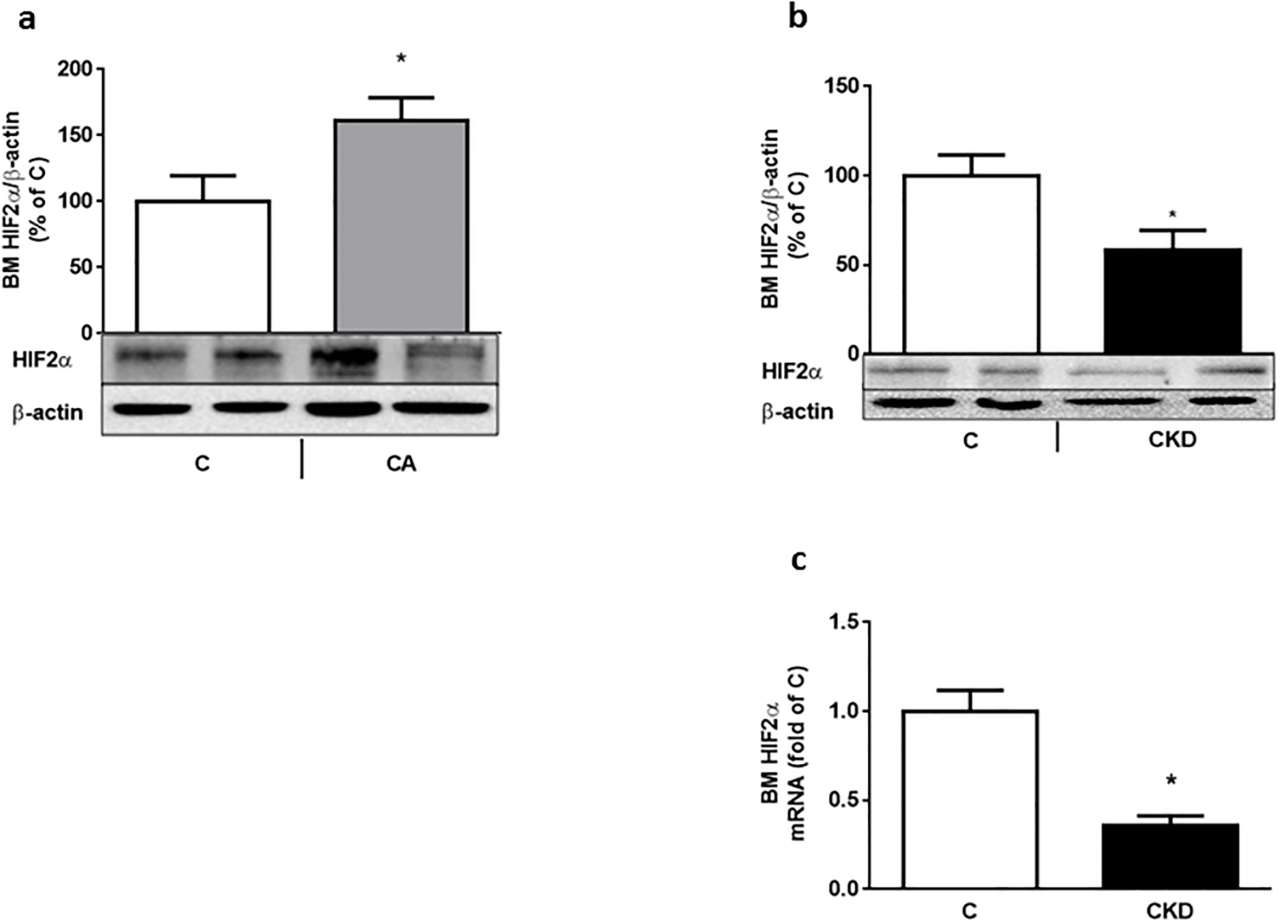
a-c: Bone marrow (BM) HIF2 α is inappropriately decreased in uremic anemia. BM HIF2 α protein (depicted as percentage of actin-corrected concentration) increases in 7 day-bled rats (Fig 2a) Vs control. Contrary to that, BM HIF2 α protein decreases in uremic (4 weeks after 5/6 nephrectomy) rats (Fig. 2b). BM HIF2 α mRNA (Fig. 2c) shows a similar pattern. * p<0.01 Vs C. 6–8 rats were used in each group.

**Fig 3:**
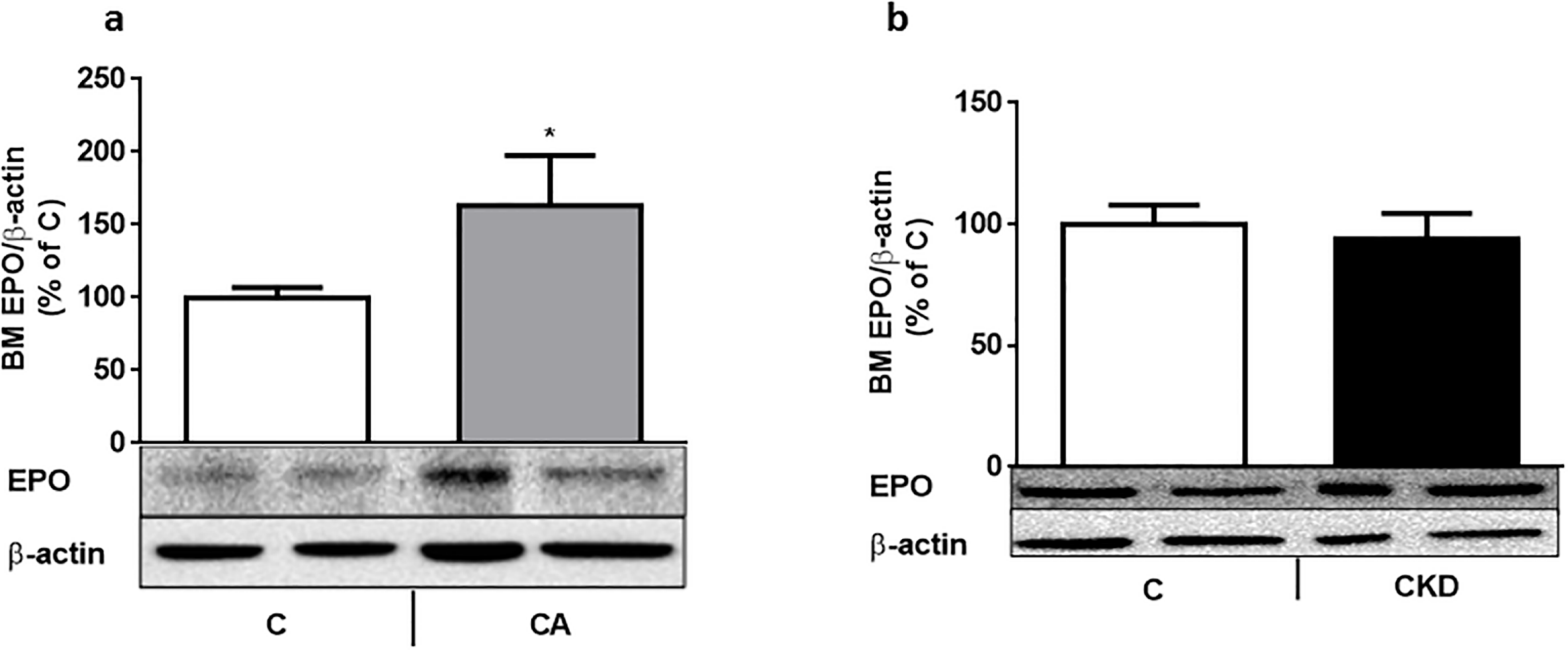
a-b: Bone marrow (BM) EPO protein levels are not increase in uremic anemic rats. BM EPO protein (depicted as percentage of actin-corrected concentration) increases in 7 day-bled rats Vs control (Fig 3a). Contrary to that, BM EPO protein does not change in uremic (4 weeks after 5/6 nephrectomy) rats (Fig 2b). The lower panel shows a representative gel. 6–8 rats were used in each group.

**Fig 4:**
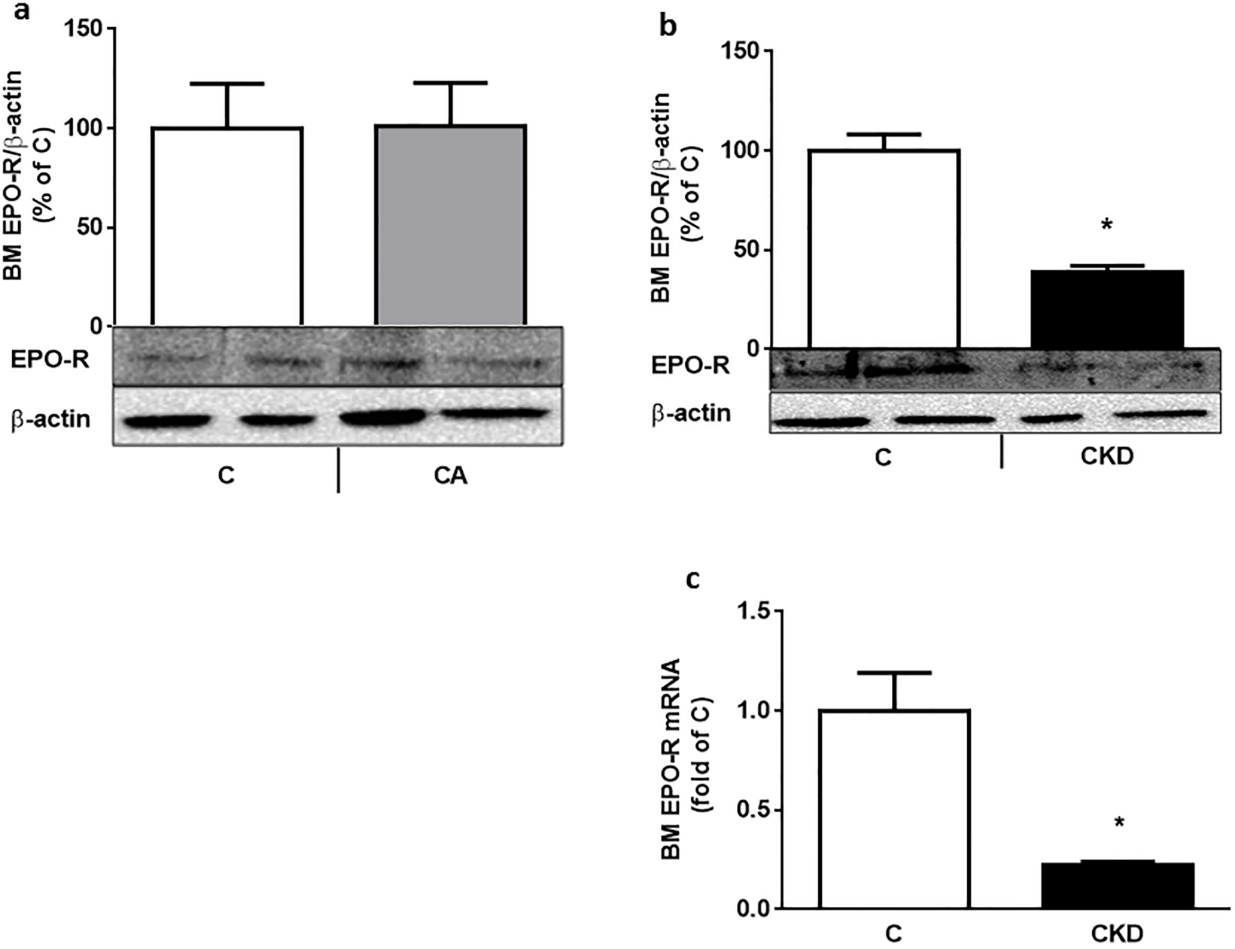
a-c: Bone marrow (BM) EPO receptor (EPO-R) decreases in uremic anemia rats. BM EPO-R protein (depicted as percentage of actin-corrected concentration) is unchanged in 7 day-bled rats Vs controls (Fig. 4a). Contrary to that BM EPO-R protein decreases in uremic (4 weeks after 5/6 nephrectomy) rats (Fig. 4b). BM EPO-R mRNA (Fig. 4c) shows a similar pattern. * p<0.01 Vs C. 6–8 rats were used in each group.

**Fig 5:**
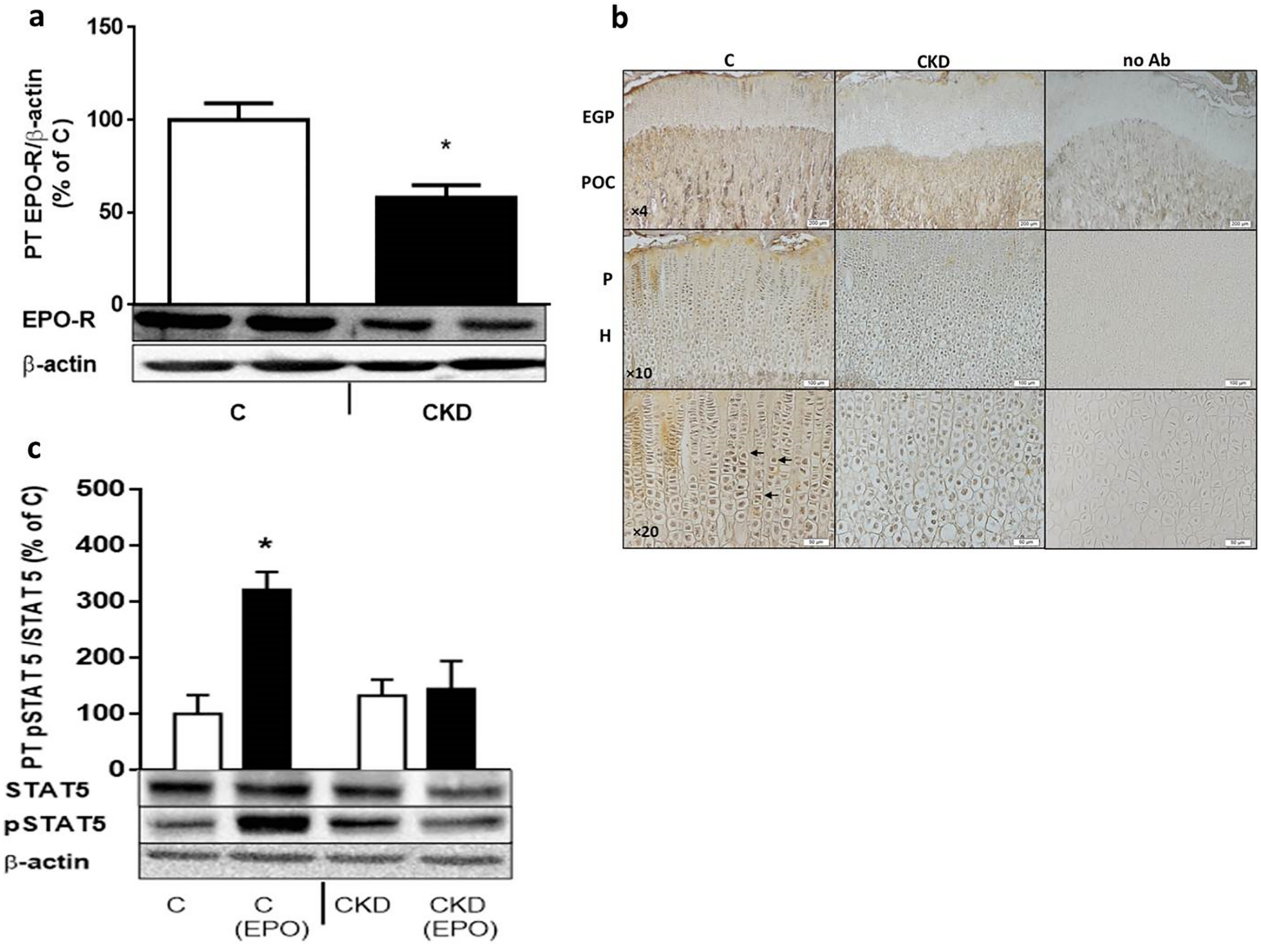
a-c: Proximal tibia (PT) EPO receptor (EPO-R) decreases in uremic rats, as shown by Western blot (Fig. 5a) and immunohistochemistry (Fig. 5b). EPO-R protein levels are depicted as a percentage of their actin-corrected concentrations). 6–8 rats were used in each group. * p<0.001 Vs C. Immunohistochemistry shows the epiphyseal growth plate (EGP) and the primary ossification center (POC). Immunostainable EPO-R appears unchanged in the POC but is decreased in the EGP, which includes the proliferative (P) and hypertrophic (H) chondrocytes. The PT lysate was assessed for EPO stimulated STAT5 phosphorylation (Fig. 5c) in control and uremic (CKD) rats. Animals received a single bolus of IV rhEPO (25 U/kg) or saline, 5 minutes prior to sacrifice. The lower panel shows a representative gel. 6–8 rats were used in each group. * p< 0.01 Vs C.

Subtotal nephrectomy induced as expected renal insufficiency: elevation of serum creatinine (1.01±0.1 Vs 0.28±0.01 mg/dL) and urea, as well as albuminuria (208.9±9.3 Vs 60.7±16.1 mg/g creatinine, p<0.001). Comparison of baseline anemia parameters between the 7-days bleeding and the subtotal nephrectomy experiments are depicted in Table 2: a similar mild anemia was induced in the blood loss group (hemoglobin concentration: 10.8±0.2 Vs 12.7±0.2 g/dL, p< 0.001), similar to the levels obtained 4 weeks after the induction of renal failure (11.4±0.3 Vs 13.5±0.3 g/dL, p< 0.001). However, hepatic hepcidin mRNA showed an opposite direction of change: contrary to a decrease after blood loss (0.32±0.1 fold of control, p<0.005), liver hepcidin mRNA increased in the CKD group (1.8±0.1 fold of C, p< 0.0001). In spite of the elevation in liver hepcidin, no changes were seen in red blood cell volume (MCV), serum iron or transferrin concentrations between C and CKD. Consistent with an appropriate response to anemia and tissue hypoxia, kidney HIF2 α protein was increased after the 7-days bleeding (187 ± 15% of C, Fig 1a). In comparison, kidney HIF-2 α was unchanged in the CKD experiment (82.8 ± 22.3% Vs 100 ± 8% in CKD and C respectively, p = NS, Fig 1b). Bone marrow HIF2 α was also increased in the bleeding experiment (161.2 ± 8.7% of C, p< 0.05, Fig 2a), but was significantly decreased in the CKD experiment, at both protein and mRNA levels (Fig 2b and 2c). No differences were seen in kidney EPO mRNA or serum EPO levels in both experiments. However, bone marrow EPO protein (which totally reflects entrapped circulating EPO) was increased in the bleeding experiment (163.3 ± 15.4% of C, p< 0.05, Fig 3a). In contrast, bone marrow EPO was unchanged in the CKD experiment (94±10.6% of C, p = NS, Fig 3b). EPO receptor was unchanged in the bleeding experiment (Fig 4a) but significantly decreased in the CKD experiment, both at the protein (38.9±3.1% of C, p< 0.001, Fig 4b) and mRNA level (22.3±1.7% of C, p< 0.001, Fig 4c). We then examined EPO-R expression and signaling in a proximal tibia (PT) samples, which included both the cartilaginous epiphyseal growth plate (EGP) and the primary ossification center (POC). EPO-R protein was similarly decreased in the CKD experiment (57.9±6.9% of C, p< 0.01, Fig 5a). Immunohistochemistry showed a similar pattern of decreased immunostainable EPO-R (Fig 5b) in CKD. Control and CKD rats’ PT EPO stimulated total and phosphorylated STAT5 were examined 5 minutes after a single 25 U/kg bolus of recombinant human EPO or saline, leading to 4 groups: C, C-EPO, CKD and CKD-EPO. This manipulation caused a significant increase in pSTAT5/STAT5 ratio in C-EPO Vs C (320.6 ± 31.9% of C, p<0.01). However, pSTAT5/STAT5 levels were unchanged in CKD Vs C or in CKD-EPO Vs CKD (Fig 5c).

**Table 2:** Basic laboratory values in the 2 experiments.

	Bleeding experiment			CKD experiment		
	C	C-A	p-value	C	CKD	p-value
N (range)	6–8	6–8		7–21	7–25	
Body weight (g)	159.5±2	149.5±2.5	< 0.01	197.3 ± 3.1	107.8 ± 6.7	<0.0001
Urea (mg/dL)	36.1±1.2	40±2.9	NS	30.6±0.9	157±10	<0.0001
Creatinine (mg/dl)	0.15±0.01	0.14±0.01	NS	0.28±0.01	1.01±0.1	<0.0001
U-albumin (mg/g)	-	-	-	60.7±16.1	208.9±9.3	<0.0001
Hemoglobin (g/dl)	12.7±0.2	10.8±0.2	<0.001	13.5±0.3	11.4±0.3	<0.001
MCV (fl)	67 ± 0.8	79.8 ± 1.2	<0.001	60.3± 0.5	63.3 ± 0.4	<0.0001
Serum iron (mg/dl)	287±16.7	350.5±35.1	0.13	149.4±6.7	168.4±9.5	0.12
Transferrin (mg/dl)	142±2.7	141.2±2	NS	149.4± 1.4	139.8±2	<0.05
Serum EPO (ng/ml)	0.12±0.02	0.14±0.01	NS	0.13±0.02	0.13±0.02	NS
Liver Hepcidin mRNA (fold of contol)	1.05±0.1	0.32±0.1	<0.001	1.1±0.07	1.8±0.1	<0.001

MCV: mean corpuscular volume. U-albumin: urine albumin, mg/gram creatinine.

## Discussion

Hypoxia is a major stimulus for HIF-2 α synthesis, which induces renal and hepatic EPO production, leading to increased serum EPO levels, stimulating then bone marrow erythropoiesis. This process needs a coordinated increase in intestinal iron absorption [18]. Intestinal lumen iron, in its ferrous form (Fe^+2^) is transported into the cytosol of enterocytes by divalent metal transporter-1 (DMT1), which is hypoxia inducible and HIF-2 regulated [19]. Absorbed iron is released from enterocytes into the circulation by ferroportin (FPN) and is then transported in complex with transferrin to liver, reticuloendothelial cells, bone marrow, and other organs. Transferrin is also HIF regulated, and hypoxia increases its serum levels. Hypoxia, low serum iron levels, and increased “erythropoietic drive” inhibit hepcidin synthesis in the liver (in a yet not fully understood pathway), resulting in diminished FPN cell surface expression in different tissues. As a result, more iron is released from enterocytes, hepatocytes, and reticuloendothelial cells. When intracellular iron levels are low, iron regulatory protein (IRP) inhibits HIF-2 translation and diminishes hypoxia-induced erythropoiesis. As shown here by us and previously by others, hepcidin is normally supposed to decrease in response to anemia and tissue hypoxia [20]. However, in CKD, hepcidin is known to be upregulated, as in other chronic inflammatory conditions [21]. Hepcidin suppression by the HIF pathway occurs indirectly through stimulation of EPO-induced erythropoiesis [22]. Hepcidin prevents intestinal iron uptake and cellular efflux by negatively modulating FPN. In spite of increased hepcidin in our CKD animals, no significant changes in serum iron or transferrin levels were seen (Table 2), probably due to the high iron content (200 mg iron/kg diet) in the "regular" rat chow provided in this experiment [23], or lack of enough time to induce iron deficiency by intestinal malabsorption. Future studies will need to address the effects of lower iron delivery or longer CKD exposure in order to investigate this aspect of uremic anemia.

In this work we show that the appropriate responses to anemia and tissue hypoxia (using repeated phlebotomies as the "gold standard") are deranged at several levels in this model of CKD in young rats: renal HIF2 α is not increased (Fig 1b), circulating as well as bone marrow EPO (which reflects circulating EPO, since no EPO is synthesized in BM [5]) is not increased. In addition, BM EPO-R expression is decreased and EPO stimulated bone STAT5 phosphorylation is inhibited. Serum EPO levels were also not increased in the bleeding experiment (Table 2). This may be due to additional factors controlling EPO synthesis in the rat, including the sympathetic nervous system [24].

EPO production by renal interstitial fibroblasts is subject to modulation by several regulators of HIF2 α including Iron Response Protein-1 [25], prolyl hydroxylases, and HIF2 α acetylases [26]. Hif-2α, one of two main Hif-α isoforms, is the critical regulator of EPO in the adult mouse. Missense mutations in HIF-2α have been shown to be a cause of erythrocytosis in humans [27]. The contribution of low grade inflammation of CKD to the inhibition of HIF2 upregulation (Figs 1 and 2) is an attractive hypothesis, as previously described in a recent model of EPO resistance in CKD rats [28]. We have also previously shown low grade inflammation in kidney tissue in a similar model of subtotal nephrectomy, manifested by elevated renal IL6, STAT3 and SOCS3 [16]. The possible correlation between CKD related low grade inflammation and HIF2 expression was shown by Suoma et al [29]. In their report, mice with prolyl hydroxylase (PHD)-deficient renal EPO producing cells also showed resistance to lipopolyscharide (LPS)-induced EPO repression in kidneys, suggesting that augmented HIF signaling counterbalances inflammatory stimuli in regulation of EPO production. Furthermore, in an animal model of inflammation induced functional iron deficiency and anemia (by use of peptidoglycan-polysaccharide), use of a HIF stabilizer (but not EPO injections) improved intestinal iron absorption and corrected anemia [30].

This lack of increase in renal and BM HIF2α in uremia naturally leads to a lack of increase in EPO levels (Fig 3). Garrido et al [31] describe an increase in circulating EPO as well as renal and liver EPO mRNA in a rat model of CKD related anemia. Anemia in this model appeared already after 3 weeks from uremia induction. However, responses to this anemic state were assessed after 12 weeks of uremia, contrary to our shorter term model (4 weeks). We were not able to show changes in renal EPO protein or mRNA, as well as circulating or bone marrow EPO. Bone marrow EPO protein levels must reflect circulating EPO as this protein is not synthesized in bone marrow [32]. In accordance to that, BM EPO was increased in our anemic control animals (Fig 3a). The anemic effects of low BM EPO levels in CKD may have been accentuated in our model by the low levels of BM EPO-R (Fig 4b and 4c). In the control anemic state EPO-R is not changed (Fig 4a). The expression of EPO-R is stringently regulated and is at a low level (1100 EPORs per primary human EPO producing cell and 300 per late-stage erythroblast) [33]. Most of the data on EPO-R regulation, including its substantial upmodulation when EPO is limited and marked down-modulation upon EPO exposure come from in vitro studies [34]. In one of our previous in vivo studies [16], renal GH receptor was also suppressed in spite of low GH availability. In spite of concerns about the reliability of current antibodies to determine EPO-R expression, we provide evidence also from mRNA (Fig 4c) and bone immunohistochemistry (Fig 5b).

The effects of low EPO and low EPO-R in bone marrow on uremic anemia are further augmented when EPO stimulated EPO-R intracellular signaling was examined: contrary to the normal expected response of increased STAT5 phosphorylation after EPO stimulation, we observed no such increase in CKD animals. A similar inhibition of GH stimulated liver JAK-STAT signaling in an animal model of uremia has been shown by Schaefer et al [35], due to an increase in SOCS2 molecules. We have also shown such upregulation of SOCS2 in bone tissue in a model of CKD related growth retardation [15]. However, a similar inhibiting molecule for EPO driven JAK-STAT signaling has not been identified. EPOR response genes include *ERFE*, *Spi2A*, and *MASL1*. As a secreted TNF related cytokine, ERFE completes a circuit between EPO action, and regulation of systemic iron levels [36].

In summary, we describe here a multilevel derangement in normal response to hypoxic-anemic signals in this rat model of CKD related anemia, both at the HIF-EPO-EPOR pathway in addition to the previously described abnormal hepcidin-iron regulatory response. The latter leads to abnormal iron absorption and mobilization and is known to be affected mostly by the known increased inflammation of uremia. The recent emergence of HIF stabilizer as means for both increases in endogenous EPO production as well as decrease in liver hepcidin [28] is a new alternative to the commonly therapeutic armamentarium that included so far mostly iron supplementation and exogenous EPO therapy. Our findings suggest that anti-inflammatory agents may play an additional role in this major CKD complication. Future studies are needed in order to analyze whether this is due to a single mechanism or as complex response to the accumulation of different uremic toxins.

## Supporting information

S1 File ARRIVE guidelines checklist (ARRIVE_checklist_Landau_0318.docx). (DOCX)

S2 File Experiments’ raw data (Lital_PLOS_raw data_0418.pdf). (PDF)

## References

[1] Fadrowski JJ, Pierce CB, Cole SR, Moxey-Mims M, Warady BA, Furth SL. Hemoglobin decline in children with chronic kidney disease: baseline results from the chronic kidney disease in children prospective cohort study. Clin J Am Soc Nephrol. 2008; 3: 457–62. doi: 10.2215/CJN.03020707 18235140 10.2215/CJN.03020707 PMC2390950

[2] Atkinson MA, Furth SL. Anemia in children with chronic kidney disease. Nat Rev Nephrol. 2011; 7: 635–41. doi: 10.1038/nrneph.2011.115 21894183 10.1038/nrneph.2011.115 PMC5739031

[3] Farsijani NM, Liu Q, Kobayashi H, Davidoff O, Sha F, Fandrey J, et al Renal epithelium regulates erythropoiesis via HIF-dependent suppression of erythropoietin. J Clin Invest. 2016; 126: 1425–37. doi: 10.1172/JCI74997 26927670 10.1172/JCI74997 PMC4811147

[4] Abed M, Artunc F, Alzoubi K, Honisch S, Baumann D, Föller M, et al Suicidal erythrocyte death in end-stage renal disease. J Mol Med (Berl). 2014; 92: 871–9. 24743961 10.1007/s00109-014-1151-4

[5] Jelkmann W. Regulation of erythropoietin production. J Physiol. 2011; 589: (Pt 6): 1251–8. doi: 10.1113/jphysiol.2010.195057 21078592 10.1113/jphysiol.2010.195057 PMC3082088

[6] Varma S, Cohen HJ. Co-transactivation of the 3' erythropoietin hypoxia inducible enhancer by the HIF-1 protein. Blood Cells Mol Dis. 1997; 23: 169–76. 9236155 10.1006/bcmd.1997.0134

[7] Gruber M, Hu CJ, Johnson RS, Brown EJ, Keith B, Simon MC. Acute postnatal ablation of Hif-2alpha results in anemia. Proc Natl Acad Sci U S A. 2007; 104: 2301–6. doi: 10.1073/pnas.0608382104 17284606 10.1073/pnas.0608382104 PMC1892942

[8] Fehr T, Ammann P, Garzoni D, Korte W, Fierz W, Rickli H, et al Interpretation of erythropoietin levels in patients with various degrees of renal insufficiency and anemia. Kidney Int. 2004; 66: 1206–11. doi: 10.1111/j.1523-1755.2004.00880.x 15327419 10.1111/j.1523-1755.2004.00880.x

[9] Lin CS, Lim SK, D’Agati V, Costantini F. Differential effects of an erythropoietin receptor gene disruption on primitive and definitive erythropoiesis. Genes Dev. 1996; 10: 154–64. 8566749 10.1101/gad.10.2.154

[10] Richmond TD, Chohan M, Barber DL. Turning cells red: signal transduction mediated by erythropoietin. Trends Cell Biol. 2005; 15: 146–55. doi: 10.1016/j.tcb.2005.01.007 15752978 10.1016/j.tcb.2005.01.007

[11] Macdougall IC, Ashenden M. Current and upcoming erythropoiesis-stimulating agents, iron products and other novel anemia medications. Adv Chronic Kidney Dis. 2009; 16: 117–30. doi: 10.1053/j.ackd.2008.12.010 19233071 10.1053/j.ackd.2008.12.010

[12] Bamgbola OF. Pattern of resistance to erythropoietin-stimulating agents in chronic kidney disease. Kidney Int. 2011; 80: 464–74. doi: 10.1038/ki.2011.179 21697809 10.1038/ki.2011.179

[13] Phrommintikul A, Haas SJ, Elsik M, Krum H. Mortality and target haemoglobin concentrations in anaemic patients with chronic kidney disease treated with erythropoietin: a meta-analysis. Lancet. 2007; 369(9559): 381–8. doi: 10.1016/S0140-6736(07)60194-9 17276778 10.1016/S0140-6736(07)60194-9

[14] Bamgbola O. Resistance to erythropoietin-stimulating agents: etiology, evaluation, and therapeutic considerations. Pediatr Nephrol. 2012; 27: 195–205. doi: 10.1007/s00467-011-1839-4 21424525 10.1007/s00467-011-1839-4

[15] Troib A, Landau D, Kachko L, Rabkin R, Segev Y. Epiphyseal growth plate growth hormone receptor signaling is decreased in chronic kidney disease-related growth retardation. Kidney Int. 2013; 84: 940–9. 23715123 10.1038/ki.2013.196

[16] Wiezel D, Assadi MH, Landau D, Troib A, Kachko L, Rabkin R, et al Impaired renal growth hormone JAK/STAT5 signaling in chronic kidney disease. Nephrol Dial Transplant. 2014; 29: 791–9. doi: 10.1093/ndt/gfu003 24463190 10.1093/ndt/gfu003

[17] Landau D, Eshet R, Troib A, Gurman Y, Chen Y, Rabkin R, Segev Y. Increased renal Akt/mTOR and MAPK signaling in type I diabetes in the absence of IGF type 1 receptor activation. Endocrine. 2009;36: 126–34. doi: 10.1007/s12020-009-9190-2 19387875 10.1007/s12020-009-9190-2

[18] Haase VH. Hypoxic regulation of erythropoiesis and iron metabolism. Am J Physiol Renal Physiol. 2010; 299:F1–13 doi: 10.1152/ajprenal.00174.2010 20444740 10.1152/ajprenal.00174.2010 PMC2904169

[19] Mastrogiannaki M, Matak P, Keith B, Simon MC, Vaulont S, Peyssonnaux C. HIF-2alpha, but not HIF-1alpha, promotes iron absorption in mice. J Clin Invest. 2009; 119: 1159–66. doi: 10.1172/JCI38499 19352007 10.1172/JCI38499 PMC2673882

[20] Means RT Jr. Hepcidin and iron regulation in health and disease. Am J Med Sci. 2013; 345: 57–60. doi: 10.1097/MAJ.0b013e318253caf1 22627267 10.1097/MAJ.0b013e318253caf1 PMC3430792

[21] Drakesmith H, Prentice AM. Hepcidin and the iron-infection axis. Science 2012; 338: 768–72. doi: 10.1126/science.1224577 23139325 10.1126/science.1224577

[22] Liu Q, Davidoff O, Niss K, Haase VH. Hypoxia-inducible factor regulates hepcidin via erythropoietin-induced erythropoiesis. J Clin Invest. 2012; 122: 4635–44. doi: 10.1172/JCI63924 23114598 10.1172/JCI63924 PMC3533545

[23] Márquez-Ibarra A, Huerta M, Villalpando-Hernández S, Ríos-Silva M, Díaz-Reval MI, Cruzblanca H, et al The effects of dietary iron and capsaicin on hemoglobin, blood glucose, insulin tolerance, cholesterol and triglycerides, in healthy and diabetic Wistar rats. PLoS One. 2016; 11:e0152625 doi: 10.1371/journal.pone.0152625 27064411 10.1371/journal.pone.0152625 PMC4827844

[24] Ditting T, Hilgers KF, Stetter A, Linz P, Schönweiss C, Veelken R. Renal sympathetic nerves modulate erythropoietin plasma levels after transient hemorrhage in rats. Am J Physiol Renal Physiol. 2007;293:F1099–106. doi: 10.1152/ajprenal.00267.2007 17634394 10.1152/ajprenal.00267.2007

[25] Mastrogiannaki M, Matak P, Peyssonnaux C. The gut in iron homeostasis: role of HIF-2 under normal and pathological conditions. Blood. 2013; 122: 885–92. doi: 10.1182/blood-2012-11-427765 23678007 10.1182/blood-2012-11-427765 PMC3743464

[26] Chen R, Xu M, Hogg RT, Li J, Little B, Gerard RD, et al The acetylase/deacetylase couple CREB-binding protein/Sirtuin 1 controls hypoxia-inducible factor 2 signaling. J Biol Chem. 2012; 287: 30800–11. doi: 10.1074/jbc.M111.244780 22807441 10.1074/jbc.M111.244780 PMC3436323

[27] Tan Q, Kerestes H, Percy MJ, Pietrofesa R, Chen L, Khurana TS, et al Erythrocytosis and pulmonary hypertension in a mouse model of human HIF2A gain of function mutation. J Biol Chem. 2013; 288: 17134–44. doi: 10.1074/jbc.M112.444059 23640890 10.1074/jbc.M112.444059 PMC3682519

[28] Ribeiro S, Garrido P, Fernandes J, Vala H, Rocha-Pereira P, Costa E, Belo L, Reis F, Santos-Silva A. Pathological and molecular mechanisms underlying resistance to recombinant human erythropoietin therapy in the remnant kidney rat model of chronic kidney disease associated anemia. Biochimie. 2016;125:150–62. doi: 10.1016/j.biochi.2016.03.012 27039028 10.1016/j.biochi.2016.03.012

[29] Souma T, Nezu M, Nakano D, Yamazaki S, Hirano I, Sekine H, et al Erythropoietin synthesis in renal myofibroblasts is restored by activation of hypoxia signaling. J Am Soc Nephrol. 2016; 27: 428–38. doi: 10.1681/ASN.2014121184 26054543 10.1681/ASN.2014121184 PMC4731118

[30] Barrett TD, Palomino HL, Brondstetter TI, Kanelakis KC, Wu X, Yan W, et al Prolyl hydroxylase inhibition corrects functional iron deficiency and inflammation-induced anaemia in rats. Br J Pharmacol. 2015; 172: 4078–88. doi: 10.1111/bph.13188 25988595 10.1111/bph.13188 PMC4543614

[31] Garrido P, Ribeiro S, Fernandes J, Vala H, Bronze-da-Rocha E, Rocha-Pereira P, Belo L, Costa E, Santos-Silva A, Reis F. Iron-hepcidin dysmetabolism, anemia and renal hypoxia, inflammation and fibrosis in the remnant kidney rat model. PLoS One. 2015; 10:e0124048 doi: 10.1371/journal.pone.0124048 25867633 10.1371/journal.pone.0124048 PMC4395008

[32] Suzuki N. Erythropoietin gene expression: developmental-stage specificity, cell-type specificity, and hypoxia inducibility. Tohoku J Exp Med. 2015; 235: 233–40. doi: 10.1620/tjem.235.233 25786542 10.1620/tjem.235.233

[33] Kuhrt D, Wojchowski DM. Emerging EPO and EPO receptor regulators and signal transducers. Blood. 2015; 125: 3536–41. doi: 10.1182/blood-2014-11-575357 25887776 10.1182/blood-2014-11-575357 PMC4458796

[34] Singh S, Verma R, Pradeep A, Leu K, Mortensen RB, Young PR, et al Dynamic ligand modulation of EPO receptor pools, and dysregulation by polycythemia-associated EPOR alleles. PLoS One. 2012; 7: e29064 doi: 10.1371/journal.pone.0029064 22253704 10.1371/journal.pone.0029064 PMC3257245

[35] Schaefer F, Chen Y, Tsao T, Nouri P, Rabkin R. Impaired JAK-STAT signal transduction contributes to growth hormone resistance in chronic uremia. J Clin Invest. 2001; 108: 467–75. doi: 10.1172/JCI11895 11489940 10.1172/JCI11895 PMC209355

[36] Kautz L, Jung G, Valore E, Rivella S, Nemeth E, Ganz T. Identification of erythroferrone as an erythroid regulator of iron metabolism. Nat Genet. 2014; 46: 678–84. doi: 10.1038/ng.2996 24880340 10.1038/ng.2996 PMC4104984

