# Supplementary material for: The hypoxia inducible factor/erythropoietin (EPO)/EPO receptor pathway is disturbed in a rat model of chronic kidney disease related anemia
Source: PLoS One. 2018 May 8;13(5):e0196684. doi: 10.1371/journal.pone.0196684 (PMC5940200; doi:10.1371/journal.pone.0196684)
Supplement: S1 File — (DOCX) [file pone.0196684.s001.docx]

March 18, 2018

Landau D et al: Erythropoietin pathway dysregulation in anemia of chronic kidney disease. PLOS One submission.

**ARRIVE Guidelines Checklist**

| **#** | **Topic** | **Recommendation** | **Checked** |
| --- | --- | --- | --- |
| 1 | Title | Accurate description of content | √ |
| 2 | Abstract | Background, objectives, methods (strain/species), results, conclusions | √ |
| 3 | Intro/Background |  | √ |
| 4 | Intro/ Objectives |  | √ |
| 5 | Methods/ Ethical statement | Ethical review permissions | √ |
| 6 | Methods/ Study design | # of groups, blinding, experimental unit | √ |
| 7 | Methods/ Experimental procedures |  | √ |
| 8 | Methods/ Experimental animals | Species, strain, sex, developmental stage | √ |
| 9 | Methods/ Housing & husbandry |  | √ |
| 10 | Methods/ sample size |  | √ |
| 11 | Methods/ Allocation to exp. Groups | (randomization not done) | √ |
| 12 | Methods/ Experimental outcomes |  | √ |
| 13 | Methods/ Statistical methods |  | √ |
| 14 | Results/ Baseline data | Health status of animals prior to testing | √ |
| 15 | Results/ Numbers analysed | # of animals used (in fig. legends) | √ |
| 16 | Results/ outcomes and estimation | Means +/- Standard errors | √ |
| 17 | Results/ adverse events |  | NA |
| 18 | Discussion/ Interpretation/ implications | Interpret the results, study limitations, sources of bias, limitations of the animal model, implications for replacement/refinement/reduction | √ |
| 19 | Discussion/ Generalisability, translation | Likelihood to translate to other species/systems, relevance to human biology | √ |
| 20 | Discussion/ Funding |  | √ |
|  |  |  |  |
