# Supplementary material for: The hypoxia inducible factor/erythropoietin (EPO)/EPO receptor pathway is disturbed in a rat model of chronic kidney disease related anemia
Source: PLoS One. 2018 May 8;13(5):e0196684. doi: 10.1371/journal.pone.0196684 (PMC5940200; doi:10.1371/journal.pone.0196684)
Supplement: S2 File — (PDF) [file pone.0196684.s002.pdf]

	CKD	C	CA	C
**Kidney HIF2a protein**	34.62	91.44	178.62	124.94
	112.80	126.95	240.76	75.73
	181.53	101.01	189.58	101.86
	22.10	132.83	177.18	97.47
	50.27	93.92	148.93	
	128.71	58.80		
	49.54	97.69		
		97.35		
**BM HIF2a**	27.28	65.72	187.16	51.94
	40.00	137.60	153.85	86.47
	73.47	74.52	150.39	120.53
	80.65	73.15	153.37	141.06
	37.51	101.51		
	92.07	106.73		
		140.77		
**BM HIF2a**	0.24	1.26		
	0.30	1.56		
	0.30	1.02		
	0.59	0.75		
	0.49	1.67		
	0.75	0.73		
	0.26	0.63		
	0.43	0.86		
	0.17	0.66		
	0.20	0.85		
	0.20			
**BM EPO**	133.60	106.78	162.15	86.74
	102.46	100.57	113.96	119.29
	109.68	93.49	182.89	99.65
	160.95	107.83	152.08	94.32
	87.84	44.87	205.45	
	49.98	119.44		
	28.54	87.34		
	80.60	90.01		
	111.34	88.86		
	73.59	77.25		
	119.26	140.29		
	70.45	143.27		
**BM EPO-R**	40.47	84.62	82.99	155.81
	42.23	99.58	87.53	83.63
	41.15	89.78	99.83	49.03
	40.02	64.81	138.27	111.54
	51.70	80.42	96.85	
	30.60	107.79		

	38.07	104.39		
	57.48	118.71		
	40.90	149.89		
	16.85			
	41.90			
	25.72			
**BM EPO-R**	0.28	0.41		
	0.18	0.48		
	0.21	1.04		
	0.21	0.41		
	0.31	2.01		
	0.22	0.57		
	0.11	1.43		
	0.21	1.24		
	0.24	1.41		
	0.30			
	0.18			
**BM EPO-R**	0.28	0.41		
	0.18	0.48		
	0.21	1.04		
	0.21	0.41		
	0.31	2.01		
	0.22	0.57		
	0.11	1.43		
	0.21	1.24		
	0.24	1.41		
	0.30			
	0.18			
**PT pSTAT5**	28.44	88.75		
	21.33	121.58		
	24.75	142.78		
	67.48	79.34		
	66.01	68.38		
	85.73	76.34		
	62.59	81.74		
	78.93	152.26		
	66.01	120.89		
	78.04	95.80		
	57.93	72.13		

		CKD	C
		164.79	34.24
		195.65	137.07
		72.50	128.69
	Untreated	95.20	
		57.29	356.84
		259.18	348.08
PT pSTAT 5	rhEPO	62.92	256.96
/STAT 5	(25 u/kg)	195.85	

	CKD	C	CA	C
Body weight (g)	122.60	184.80	158.00	167
	102.10	180.50	145.00	149
	142.80	189.00	154.00	157
	122.20	200.70	147.00	164
	80.80	188.50	136.00	159
	145.50	183.50	150.00	160
	113.60	182.50	149.00	156
	153.50	193.60	157.00	164
	77.50	186.50		
	190.20	192.70		
	90.10	218.70		
	66.60	211.30		
	102.20	215.40		
	87.90	177.60		
	97.70	203.70		
	52.70	226.20		
	51.10	221.10		
	130.20	200.30		
	112.90	197.10		
	123.60	189.90		
	132.70	199.80		
	109.10			
	53.80			
	117.10			
	117.40			
Urea (mg/dL)	92.08	27.75	34.10	38.40
	176.76	31.66	31.50	39.50
	116.20	36.59	51.00	32.70
	112.51	31.61	43.90	35.20
	91.35	34.06	42.20	32.50
	92.82	29.60	37.30	38.00
	221.50	26.10		
	241.36	33.20		
	184.47	26.83		
	233.22	28.25		
	125.72	30.78		
	146.54	37.76		
	139.51	36.76		
	195.32	28.72		
	139.29	26.55		
	141.56	29.77		
	145.99	27.70		
	160.49	30.56		
	133.35	26.44		
	180.42			
	225.61			

Creatinine (mg/dl)	0.70	0.25	0.19	0.14
	1.45	0.33	0.14	0.13
	0.74	0.27	0.13	0.15
	0.69	0.25	0.13	0.15
	0.69	0.25	0.12	0.16
	0.65	0.29	0.15	0.15
	1.34	0.23		
	1.49	0.29		
	1.05	0.22		
	1.42	0.31		
	0.75	0.36		
	0.85	0.33		
	0.98	0.34		
	1.56	0.27		
	0.91	0.33		
	0.83	0.22		
	1.11	0.28		
	0.97	0.24		
	0.68	0.44		
	0.88			
	1.55			
U-albumin (mg/g)	237.56	51.27		
	247.87	131.17		
	247.26	6.78		
	233.54	41.66		
	234.89	12.99		
	153.73	150.51		
	181.58	35.60		
	245.91	141.54		
	228.83	18.78		
	115.92	38.83		
	243.08	38.93		
	207.48			
	210.44			
	249.50			
	194.34			
	177.35			
	195.29			
	155.56			
	11.00			
Hgb (g/dl)	13.00	14.10	11	12.6
	13.40	14.20	10.7	12.8
	11.00	13.50	11.1	11.9
	11.50	12.20	11.3	13.1
	11.80	13.20	10.5	13.1
	8.00	12.70	9.8	12.7
	9.80	13.40	11	12.2
	12.00	14.10	11.2	12.9
	11.60	13.90		
	11.30	9.80		

	12.30	14.20		
	11.60	13.80		
	10.50	13.30		
	11.90	14.80		
	11.80	14.60		
	64.50	14.60		
MCV (fl)	63.40	62.40	78.1	65.3
	65.90	58.50	77.2	68.3
	60.70	61.20	79.9	64.1
	63.70	59.80	78.4	68.2
	62.90	58.40	85.9	71
	63.10	59.70	75.2	65.8
	62.70	57.60	80.8	67
	63.70	58.20	82.6	66.2
	61.50	60.40		
	64.90	64.80		
	63.80	58.70		
	59.30	61.20		
	63.90	59.20		
	65.90	59.80		
	63.00	61.20		
		61.10		
		62.30		
Serum iron (mg/dl)	232.00	152.00	208.00	306.00
	138.00	230.00	399.00	295.00
	204.00	150.00	369.00	241.00
	133.00	121.00	440.00	245.00
	128.00	151.00	289.00	351.00
	150.00	148.00	398.00	284.00
	131.00	182.00		
	169.00	121.00		
	156.00	173.00		
	230.00	129.00		
	144.00	139.00		
	163.00	100.00		
	184.00	176.00		
	196.00	165.00		
		159.00		
		133.00		
		137.00		
		114.00		
	146.65	159.00		
	134.88	154.77	143.00	144.00
	127.97	163.48	141.00	139.00
	127.79	142.44	145.00	134.00
	146.33		134.00	141.00
	143.64	149.04	137.00	140.00
	129.64	147.25	147.00	154.00
	138.40	147.50		
	131.15	153.00		

**Transferrin (mg/dl)**	132.99	150.09		
	149.53	146.19		
	137.16	144.36		
	135.20	142.99		
	161.60	148.01		
	151.21	150.72		
	144.35	153.57		
	131.81	137.86		
	133.45	152.67		
	151.40	154.79		
	140.83	150.64		
	0.36			
**Liver Hepcidin mRNA**	1.44	1.08	0.37	0.83
	1.55	1.18	0.02	0.79
	2.41	0.76	0.03	0.68
	2.41	0.78	0.98	1.36
	1.83	0.75	0.22	1.37
	1.55	1.53	0.24	1.35
	1.54	1.38		
	0.99	1.42		
	2.05			
	2.26			
**Serum EPO**	0.183	0.075	0.168	0.132
	0.071	0.18	0.078	0.185
	0.105	0.095	0.105	0.085
	0.135	0.123	0.073	0.071
	0.216	0.195	0.126	0.077
	0.092	0.088	0.147	0.102
	0.077	0.132	0.267	0.164
